# Secreted frizzled-related protein 3 is associated with metabolic disturbances in gestational diabetes

**DOI:** 10.3389/fendo.2026.1905877

**Published:** 2026-08-14

**Authors:** Thor Ueland, Annika Elisabet Michelsen, Elisabeth Qvigstad, Marie Cecilie Paasche Roland, Ane Cecilie Westerberg, Trond Melbye Michelsen, Pål Aukrust, Jens Bollerslev, Tove Lekva

**Affiliations:** 1 Research Institute of Internal Medicine, Oslo University Hospital, Rikshospitalet, Oslo, Norway; 2 Institute of Clinical Medicine, Faculty of Medicine, University of Oslo, Oslo, Norway; 3 Dept of Endocrinology, Morbid Obesity and Preventive Medicine, Oslo University Hospital, Oslo, Norway; 4 Department of Obstetrics, Division of Obstetrics and Gynecology, Oslo University Hospital Rikshospitalet, Oslo, Norway; 5 Department of Medical Biochemistry, Oslo University Hospital Rikshospitalet, Oslo, Norway; 6 Section of Clinical Immunology and Infectious Diseases, Oslo University Hospital, Oslo, Norway

**Keywords:** aortic stiffness, Dickkopf-1, gestational diabetes, secreted frizzled-related protein-3, Wnt signaling

## Abstract

**Aims:**

Secreted modulators of the Wingless (WNT) pathways are associated with cardiometabolic dysregulation. Among these, Dickkopf-1 (DKK1) and secreted frizzled-related protein-3 (sFRP3) are abundantly expressed in placental tissue and secreted into the maternal circulation. We hypothesized their plasma levels would be dysregulated in women with gestational diabetes mellitus (GDM) and correlate with indices of metabolic health.

**Methods:**

A total of 284 women during pregnancy and at 5 years postpartum participated in this longitudinal cohort study. Secreted Wnt modulators were analyzed by enzyme immunoassay 4 times during pregnancy and postpartum. Lipids and indices of glucose tolerance were assessed twice in pregnancy and at follow-up. Body composition and pulse wave velocity analysis was assessed at follow-up.

**Results:**

Our main findings: i) plasma DKK1 was lower throughout pregnancy in women with GDM. ii) sFRP3 was lower early in pregnancy in women with GDM and correlated with indices of obesity and adipose tissue related inflammation, insulin resistance, poor β-cell function and unfavorable lipid ratios. iii) low DKK1 during pregnancy was associated with higher aortic stiffness at follow-up.

**Conclusions:**

Low plasma sFRP3 during early pregnancy is associated with metabolic and inflammatory dysregulation while persistently low DKK1 may be associated with future cardiovascular disease risk in GDM pregnancies.

## Introduction

Gestational diabetes mellitus (GDM) refers to glucose intolerance diagnosed during pregnancy where pancreatic β-cells produce inadequate insulin for increased needs during pregnancy, in particular during its late phase (1), potentially exposing a predisposition to decreased insulin sensitivity and glucose intolerance. As the most prevalent metabolic disorder in pregnancy, GDM affects approximately 1 in 7 live births worldwide, with an estimated global prevalence of 14% (2). Hence, a diagnosis of GDM is associated with higher risk of metabolic complications during pregnancy, as well as future type 2 diabetes mellitus (T2DM), an established risk factor for cardiovascular disease (CVD) (3). While GDM is typically diagnosed at 24–28 weeks of gestation and earlier detection lacks diagnostic accuracy, glucose intolerance is manifest in all trimesters of pregnancy (4). Also, an unfavorable cardiometabolic profile characterized by dysregulated lipid levels, higher indices of adiposity and adipose tissue inflammation are present early in GDM pregnancies (5–7) indicating that early maternal metabolic alterations precede a diagnosis of GDM.

The underlying mechanisms of GDM are unclear, but involve the diabetogenic effect of hormones produced by the placenta (8). GDM has also been linked to impaired placental development (9, 10) and placental villi isolated from women with GDM display dysregulated expression of proteins involved in glucose metabolism, calcium- handling, inflammation and nutrient transport (11, 12). Among signaling pathways of importance for placental development, the Wingless (WNT) pathways play a major role as a developmental pathway controlling diverse cellular processes within the placenta such as proliferation, survival, cell fate determination and migration (13). The majority of Wnt ligands and Frizzled receptors have been detected in first trimester placental tissue (14). Furthermore, this pathway is linked to both T2DM (15), obesity induced inflammation and lipid metabolism (16). Indeed, transcription factor 7-like 2 (TCF7L2), a key transcriptional effector of the Wnt/β-catenin signaling pathway and a T2DM candidate gene has been linked to GDM, interacting with adiposity and insulin secretion (17). The Wnt proteins initiate signaling by binding receptor complexes composed of a Frizzled receptor and a low-density lipoprotein receptor-related protein 5/6, resulting in different intracellular responses, involving β-catenin (canonical pathway), Ca^2+^ or other second messengers (non-canonical pathways). Activation of Wnt/β-catenin signaling by Mucin1 impaired glucose uptake and promoted apoptosis and trophoblast cell dysfunction in GDM placentas (18) but so far there are limited studies investigating Wnt signaling in GDM.

A number of endogenous secreted pathway modulators, such as Dickkopfs (DKKs) and the secreted frizzled-related proteins (sFRPs), may regulate both canonical and non-canonical Wnt signaling by binding various ligands and receptors (19). Mechanistically, these modulators regulate Wnt signaling through distinct extracellular interactions. DKK1 and SOST act as antagonists by binding to LRP5/6 co-receptors, thereby blocking canonical pathway activation. sFRP3 inhibits signaling by directly sequestering Wnt ligands or binding to Frizzled receptors. Conversely, RSPO3 acts as an agonist by stabilizing Frizzled receptors, which enhances Wnt signal transduction (20, 21). Furthermore, these modulators often circulate at readily measurable levels and may represent a negative feedback loop to limit Wnt pathway activation, thereby reflecting activity in the Wnt signaling pathways. Using a 4-vessel sampling method at scheduled cesarean delivery, enabling us to calculate the levels of placenta-derived proteins, we recently demonstrated that several Wnt modulators were abundantly secreted by the placenta into the maternal circulation including the secreted Wnt antagonists DKK1, sFRP3 and sclerostin (SOST) and the Wnt agonist R-spondin 3 (RSPO3) (22). We and others have shown increased circulating DKK1 and SOST in prediabetes and T2DM, and that these proteins are associated with glycemic indices, lipid metabolism and obesity (23–26). Fewer studies have evaluated sFRP3 and RSPO3, but reduced skeletal muscle sFRP3 levels in T2DM were associated with inflammation and insulin resistance (27) and RSPO3 has been shown to influence body fat distribution and adipocyte biology in experimental models (28).

Based on the emerging role of Wnt signaling in metabolic disease and the potential placental contribution to the maternal circulation of secreted Wnt modulators, we hypothesized that plasma levels of DKK1, sFRP3, SOST and RSPO3 would be dysregulated in GDM. We therefore evaluated plasma levels of these in 284 women from a prospective cohort study at multiple times during pregnancy and at 5-year follow-up. We evaluated associations with adiposity, indices of glucose metabolism, unfavorable lipid ratios and arterial stiffness.

## Materials and methods

### Study population

**Figure 1:**
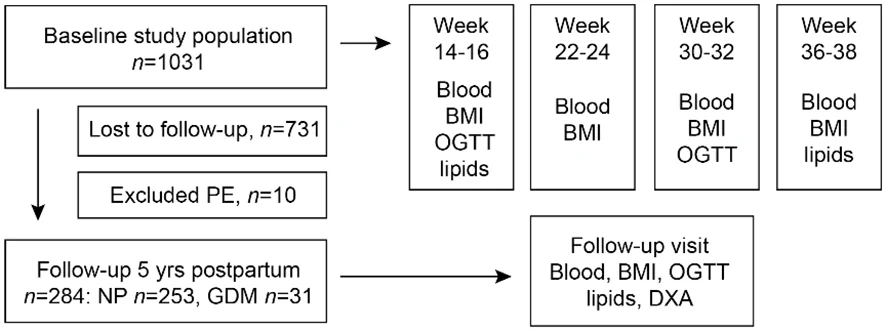
Study design. PE, preeclampsia; NP, normal pregnancy; GDM, gestational diabetes mellitus; BMI, body mass index; OGTT, Oral Glucose Tolerance Test; DXA, Dual-Energy X-ray Absorptiometry.

The STORK study is a prospective longitudinal cohort study where 1031 low-risk women of Scandinavian heritage were followed throughout their pregnancy and gave birth at Oslo University Hospital, Rikshospitalet between 2002 and 2008 (29). Exclusion criteria were multiple pregnancies, known pregestational diabetes and any severe chronic disease. Each participant had 4 study-related antenatal visits at weeks 14-16, 22-24, 30-32, and 36-38. A 75 g oral glucose tolerance test (OGTT) was performed in all women at 14–16 and again at 30–32 weeks of gestation. All women were invited to participate in a 5-year postpartum follow-up study, and 300 from the original study agreed (30). In the present study, we included only the women who had participated both during pregnancy and follow-up visits. Women with preeclampsia were excluded, and this study ended up with 284 participants with available blood samples. Subscapular subcutaneous fat was estimated during pregnancy using a Holtain caliper (Holtain, Crymych, UK) (31). The study design and measurement performed at different visits is shown in **Figure 1**.

Written informed consent was obtained from all study participants. All clinical investigations were conducted in accordance with the principles enshrined in the Declaration of Helsinki. The study was approved by the Regional Committee for Medical Research Ethics of Southern Norway in Oslo, Norway (REK S-01191 and REK S-07392a).

### Measurements of glycemic and lipid parameters

All 75 g OGTTs were performed in the morning after an overnight fast. Venous blood samples were allowed to clot for 30 min and the serum separated by centrifugation for 10 min at 3000g and stored at −80 °C. Glucose levels were measured from frozen serum samples collected at 14–16 and 30–32 weeks using the hexokinase method (Hitachi Modular P800, Roche Diagnostics, Mannheim, Germany) at an accredited clinical chemistry laboratory at Oslo University Hospital Rikshospitalet, as reported (30). While immediate glycolysis inhibition using fluoride-oxalate plasma is preferred for OGTTs, serum separated within 2 hours was preferred over EDTA plasma, as this matrix was also preferred for Insulin measurement. For the 5-year follow-up study, we used the glucose data from the Accu-check Sensor glucometer (Roche Diagnostics, Mannheim, Germany). Insulin levels in the stored serum samples were assayed in duplicate by radioimmunoassay (Diagnostic Products Corporation, Los Angeles, CA, USA), as reported (30).

Lipids were measured at an accredited laboratory at Oslo University Hospital, Rikshospitalet on a Hitachi Modular P800 (Roche Diagnostics). For this study we focused on the atherogenic triglyceride (TG)/high density lipoprotein cholesterol (HDL) ratio, identified as a risk factor for CVD in hypertensive populations (32) and which we have previously shown increased in this population (6). These were measured at 14-16- and 36-38-weeks during pregnancy. For correlation purposes, the TG/HDL ratio determined at 14–16 weeks was used both at week 14–16 and 22–24 and the TG/HDL ratio determined at 36–38 weeks was used at week 30–32 and 36-38. The TG/HDL ratio was also assessed at 5-years follow-up.

### Diagnosis of GDM

GDM was diagnosed after a 75 g OGTT using the WHO criteria from 1999: fasting glucose ≥7.0 mmol/l and/or 2 h plasma glucose ≥7.8 mmol/L (33) as these criteria are associated with more severe metabolic impairment and cardiovascular risk (6). Estimation of insulin sensitivity was measured on the same serum samples collected at the time of OGTT using the Matsuda index (i.e., 10, 000/√[(fasting glucose (mmol/L) × fasting insulin (mU/L)) × (mean glucose (mmol/L) × mean insulin (mU/L))]) during OGTT. Estimation of β-cell function was assessed with the insulin secretion-sensitivity index (ISSI-2) (area under the curve (AUC) insulin (mU/L)0–120/glucose (mmol/L)0–120 × Matsuda). Estimation of homeostasis model assessment: insulin resistance (HOMA–IR) was calculated as fasting insulin (mU/L) × fasting glucose (mmol/L)/22.5. OGTTs were performed at week 14–16 and 30-32. For correlation purposes, the above glycemic indexes determined at the 14–16 week sample were used at both week 14–16 and 22–24 and the 30–32 week sample was used at week 30–32 and 36-38.

### Measurement of inflammatory parameters and secreted Wnt proteins

Plasma levels of DKK1 (Cat# DY1906), sFRP3 (Cat# DY192), SOST (Cat# DY1406) and RSPO3 (Cat# DY3500) were measured at all antenatal visits and 5-year follow-up in EDTA plasma that was sampled on ice, centrifuged for 25 min at 3000g at 4 °C to obtain platelet-poor plasma, separated, and stored at −80 °C until analysis by enzyme immunoassays (EIA) obtained from R&D Systems (Minneapolis, MN). EDTA plasma was specifically chosen for Wnt modulators because serum is unsuitable for these analytes; platelets contain abundant pools of proteins like DKK1, and degranulation during blood clotting would artificially elevate levels and mask true *in vivo* systemic concentrations (34). Serial samples from a given individual were analyzed on the same plate. Plasma levels of leptin (Cat# DY398), total adiponectin (Cat# DY1065) and CRP (Cat# DY1707) have been reported previously (5), and were determined by EIAs from R&D Systems. All EIAs were performed in a 384-format using a combination of a SELMA pipetting robot (Analytik Jena GmbH, Jena, Germany) and a BioTek dispenser/washer (BioTek, Agilent Technologies Inc., Santa Clara, CA). Absorption was read at 450 nm with wavelength correction set to 540 nm using an EIA plate reader (BioTek). The intra- and inter-assay coefficients of variation from our laboratory data were less than 10% for all measurements.

### Measurements of arterial stiffness

Arterial stiffness was assessed by means of pulse wave velocity (PWV) measurements of the carotid and femoral arteries using SphygmoCor (Atcor Medical, Sydney, Australia), a non-invasive technique with direct-contact pulse sensors, and the method and results have previously been published (6).

### Measurements of body fat composition

Total body composition was determined by dual-energy X-ray absorptiometry (DXA; GE Lunar Prodigy Densitometer, software version 12.10, GE Medical Systems, Lunar Corp., Madison, WI, USA) and analyzed using enCORE software (version 14.10; GE Medical Systems) to determine visceral (VAT) and subcutaneous adipose tissue (SAT), as previously described (30).

### Evaluation of Wnt family expression from public databases

mRNA expression of secreted Wnt modulators were obtained from the protein atlas (proteinatlas.org). We used data from a study (GSE5999) assessing global mRNA expression from basal plate biopsy specimens obtained from 36 placentas (14–40 weeks) at the conclusion of normal pregnancies (35, 36) and a study (GSE121974) assessing whole blood global mRNA expression changes with gestational age in normal pregnancy (37).

### Statistical analysis

Data are expressed as mean ± SD when normally distributed and median (25th, 75th percentile) when skewed. Comparison of demographic data were assessed using t test or Mann–Whitney U depending on distribution, and Chi square test for categorical variables. Several measures such as glycemic, lipid, inflammatory and atherogenic parameters were compared by multivariate general linear model (GLM) with age and BMI as covariates.

Comparison of secreted Wnt proteins during pregnancy between women with and without a history of GDM was performed using a generalized linear mixed model (GLMM) with untransformed protein levels and a log link function to assess the impact of GDM over time. The model included fixed effects of GDM, time, their interaction (GDM*time), with age and BMI as covariates. BMI assessed at each time-point was used. Random effects accounted for individual variability among individual participants. We performed *post-hoc* analyses using sequential Sidak correction. A similar model was used when assessing secreted Wnt proteins during pregnancy in relation to dichotomized PWV at 5-year follow-up.

Multivariable logistic regression analysis was performed to evaluate the independent predictive value of Wnt antagonists at 14–16 weeks for GDM risk. Two separate models were constructed, both adjusting for the strongest clinical indices identified in the Receiver Operating Characteristic (ROC) analysis (ISSI2 and TG/HDL ratio). Model 1 evaluated the independent effect of DKK1, while Model 2 evaluated sFRP3. Results are presented as odds ratios (ORs) with 95% confidence intervals (CIs).

Plasma levels of protein at 5 years follow-up in relation to previous GDM status was assessed by multivariate GLM with proteins as dependent, GDM status as fixed and age and sex as covariates. Correlations between secreted Wnt modulators and glycemic, lipid, inflammatory and atherogenic parameters as well as correlation between mRNA expression of Wnt family members and gestational age at sampling were assessed by Spearman correlation. Due to the exploratory nature of the temporal and multiple correlation analyses, p-values were not adjusted for multiple comparisons. Two-tailed p values <0.05 were considered significant.

## Results

**Table 1** shows the characteristics of the study population during pregnancy and at 5-year follow-up stratified into women with a normal pregnancy (NP) and those with GDM in the index pregnancy (31 with and 253 without GDM) using the WHO 1999 criteria based on OGTTs. The parent STORK cohort originally included 1031 participants, of whom 300 returned at 5 years and 284 were included in the current analysis. Baseline evaluation indicated that GDM status, BMI, and examined metabolic characteristics did not differ significantly between participants included in the follow-up and those lost to follow-up, however selection bias related to unmeasured characteristics cannot be completely excluded.

**Table 1:** Characteristics of the study population during pregnancy and at 5-year follow-up.

Clinical variables	Visit 3 (week 30–32) in the index pregnancy		Follow-up visit	
	NP (n=253)	GDM (n=31)	NP (n=253)	GDM (n=31)
Follow-up time (years)			4.8 (4.4, 5.4)	5.0 (4.5, 5.4)
Age (years)	32.2 ± 3.8	33.1 ± 3.7	37.5 ± 3.8	38.6 ± 3.8
Primipara n (%)	122 (48.6)	18 (60.0)	26 (10.3)	6 (19.3)
Family history CVD			143 (57.0)	22 (75.9)*
Family history diabetes			75 (29.6)	13 (41.9)
Curr./prev. smoking %	3/18	3/26	15/23*	29/26
Systolic BP (mmHg)	112 ± 11	111 ± 10	112 ± 11	114 ± 15
Diastolic BP (mmHg)	68 ± 9	67 ± 6	69 ± 9	71 ± 10
Adiposity				
BMI (kg/m^2^)	26.7 ± 3.8	28.2 ± 4.6*	23.5 ± 3.8	25.2 ± 5.1*
SFT subscapular (mm)	21 (14, 25)	26 (18, 35)		
VAT mass (g)			177 (77, 372)	393 (150, 900)***
SAT mass (kg)			1.1 (0.8, 1.5)	1.5 (1.0, 1.8)
Glucose metabolism				
Insulin sensitivity	118 (83, 179)	64 (49, 100)***	249 (178, 339)	189 (111, 274)**
Insulin resistance	1.1 (0.7, 1.7)	1.9 (1.2, 2.6)***	0.7 (0.4, 1.0)	1.0 (0.5, 1.3)
β-cell function	969 (729, 1253)	590 (444, 679)***	1051 (832, 1386)	712 (559, 1247)***
Inflammation				
CRP	1.4 (0.8, 2.2)	1.9 (1.0, 2.7)	0.4 (0.2, 0.7)	0.6 (0.3, 1.4)
Leptin/adiponectin ratio	7.4 (4.8, 11.4)	9.8 (5.2, 15.7)**	3.1 (1.8, 5.9)	4.3 (2.9, 10.8)*
Atherogenic indices				
PWV (ms)			6.6 (6.1, 7.1)	6.9 (6.4, 7.3)*
TG/HDL ratio	0.9 (0.5, 1.3)	1.2 (0.8, 1.8)**	0.45 (0.36, 0.62)	0.65 (0.46, 1.02)***

Data given as mean ± SD when normal distributed and median (25th, 75th) when skewed distributed. Categorical data are expressed as n (%). Comparison between women with GDM and normal pregnancy were performed using t test for normal distributed variables, Mann–Whitney U for non-distributed continuous variables, and Chi test for categorical variables. However, for measures of adipocity, glucose metabolism, Inflammation and atherogenic indices, multivariate GLM was used with age and BMI as covariates. *p < 0.05, **p <0.01, ***p < 0.001. BMI, body mass index; BP, blood pressure; CRP, C-reactive protein; GDM, gestational diabetes mellitus; HDL, high-density lipoprotein cholesterol; PWV, pulse wave velocity; SAT, subcutaneous adipose tissue; SFT, skinfold thickness; TG, triglyceride; VAT, visceral adipose tissue.

Women with GDM had a higher BMI during pregnancy and at follow-up and more history of CVD in their family. We included several previously reported measures to correlate with secreted Wnt modulator levels and assessed these in models adjusting for age and BMI. Thus, women with previous GDM had a higher VAT mass and indices of glucose metabolism were dysregulated during pregnancy and at 5 years follow-up (30). Similarly, the leptin/adiponectin ratio (5) and TG/HDL (6) ratios were higher in GDM during pregnancy and at follow-up. Aortic stiffness was higher at follow-up in women with previous GDM (6).

### Secreted Wnt modulators during pregnancy

**Figure 2:**
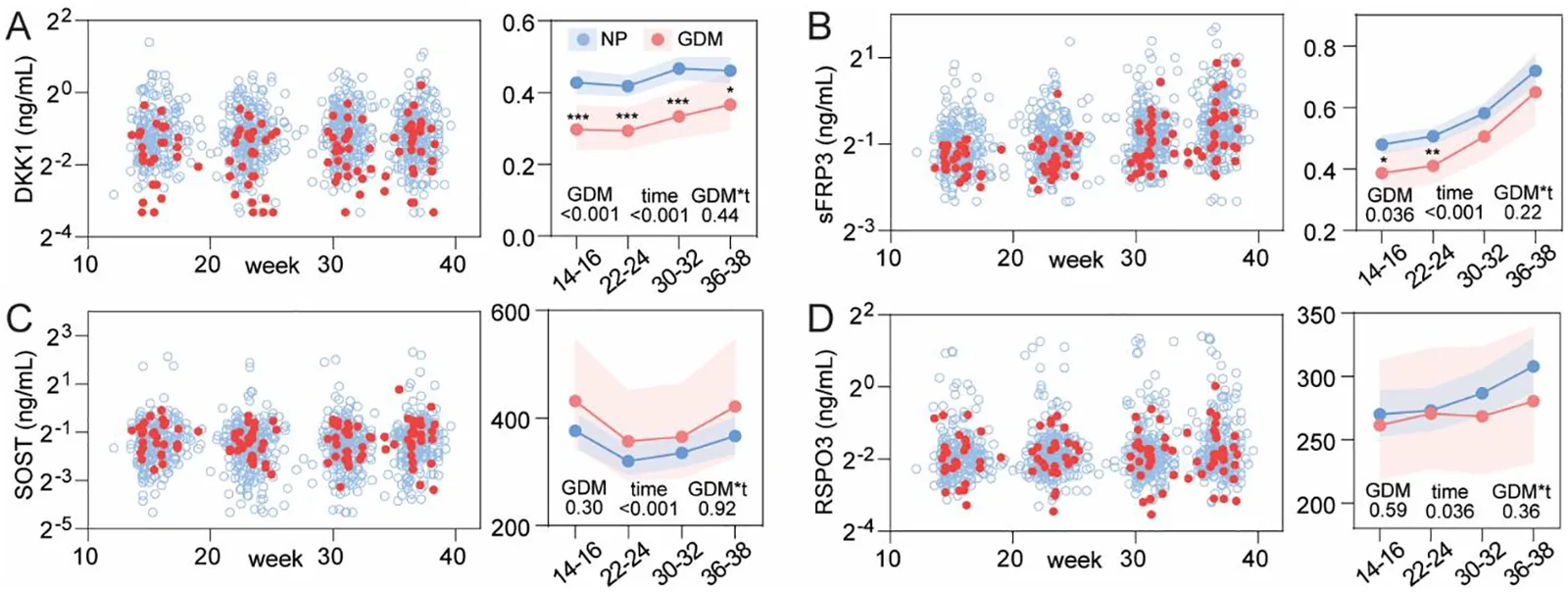
Circulating Wnt modulators in gestational diabetes. Plasma levels of **(A)** Dickkopf-related protein 1 (DKK1) **(B)** secreted frizzled related protein 3 (sFRP3) **(C)** sclerostin (SOST) and **(D)** R-spondin 3 (RSPO3) in pregnancy with gestational diabetes mellitus (GDM, red) and normal pregnancy (NP, blue). The left dot plot in each panel shows protein levels according to gestational week while the right graph shows estimated marginal means and 95% confidence intervals (shaded) from a linear mixed model analysis with age and BMI as covariates. The group effect (GDM), time effect and group*time interaction p-values are shown. *p<0.05, **p<0.01, ***p<0.001 between groups at given time-point.

As shown in **Figure 2A**, DKK1 was lower in GDM at all time-points and displayed a modest increase during pregnancy. Also, sFRP3 was lower in women with GDM in early pregnancy followed by a pronounced increase towards term (**Figure 2B**). No differences regarding GDM status were observed for SOST and RSPO3 (**Figures 2C, D**), and these were excluded from further analysis.

### Low sFRP3 early in GDM pregnancies is associated with metabolic dysregulation

**Figure 3:**
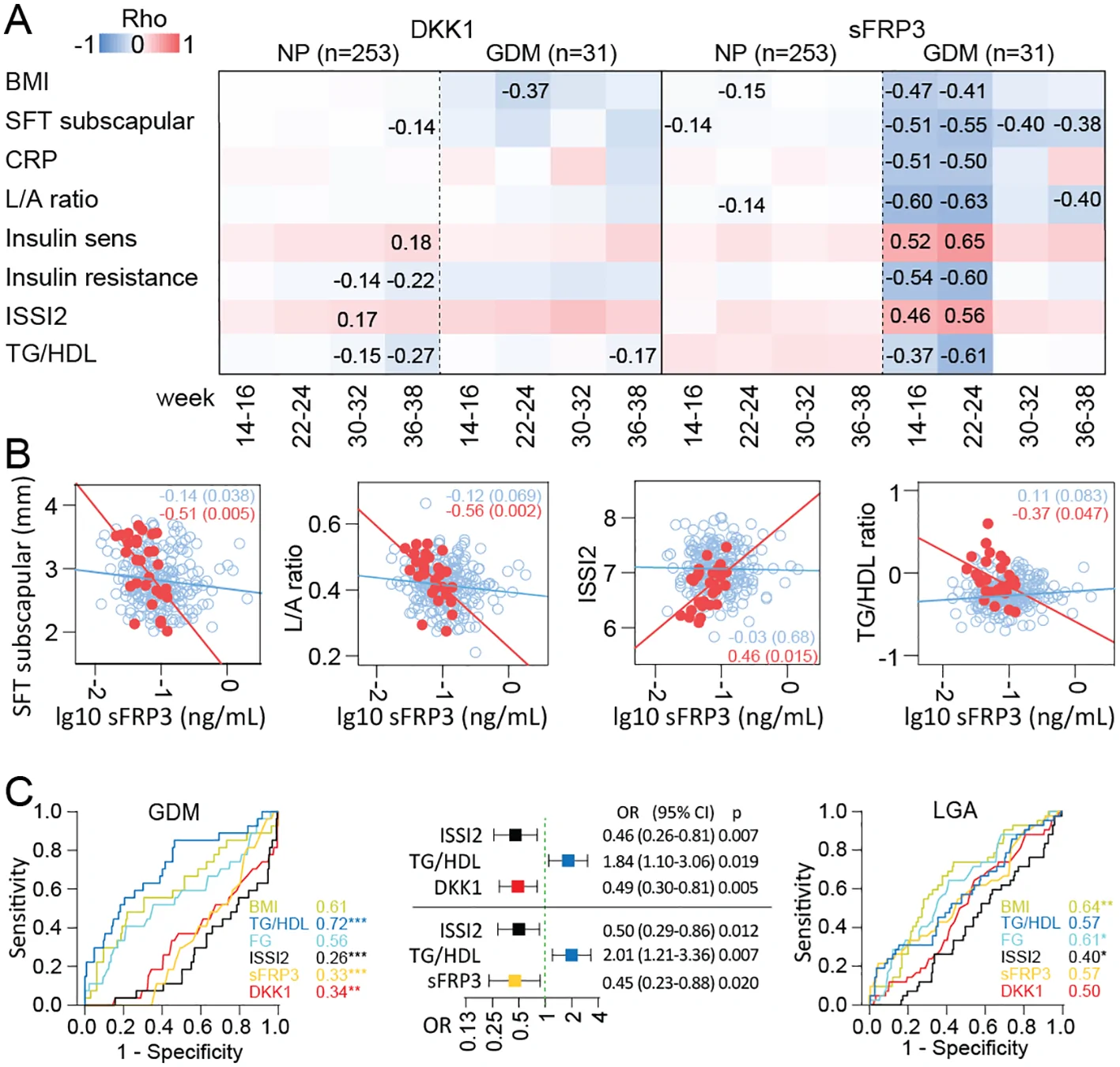
Associations between circulating Wnt modulators and metabolic indices: predictive value of early-pregnancy Wnt antagonists. **(A)** Heatmap showing correlation (spearman, significant correlations are indicated by numbers (rho)) between plasma Dickkopf-related protein 1 (DKK1), secreted frizzled related protein 3 (sFRP3) and measures of adiposity (BMI, body mass index; SFT, skinfold thickness), inflammation (CRP, C-reactive protein; L/A ratio, leptin/adiponectin), glucose metabolism (insulin sensitivity, Matsuda index; insulin resistance, HOMA-IR; β-cell function, insulin secretion-sensitivity index-2 (ISSI2)) and atherogenic lipid ratios (LDL/HDL, low-density lipoprotein/high-density lipoprotein; TG/HDL, triglycerides/HDL) in normal pregnancy (NP) and gestational diabetes mellitus (GDM) at different time-points. **(B)** selected correlations between sFRP3 and different indices at 14–16 weeks in NP (blue) and GDM (red). Numbers are rho with p-value in parentheses. **(C)** Receiver operating characteristic (ROC) curve and logistic regression analyses for GDM and large-for-gestational-age (LGA) infant prediction at 14–16 weeks. ISSI2, sFRP3 and DKK1 are low in GDM and AUC values below 0.5 and Odds Ratios (OR) below 1 reflect an inverse association, with lower biomarker levels indicating greater GDM probability. Left: ROC curves for GDM including TG/HDL, ISSI2, BMI, fasting glucose (FG), and Wnt antagonists. Middle: Forest plot illustrating two individual logistic regression models containing either DKK1 or sFRP3, both adjusted for ISSI2 and TG/HDL. Right: ROC curves for LGA utilizing the same clinical parameters and Wnt antagonists.

We next assessed if levels of DKK1 and sFRP3 correlated with metabolic features during pregnancy. As shown in **Figure 3A**, some modest correlations with glycemic indexes and the TG/HDL ratio were observed for DKK1 towards the end of pregnancy in women without GDM. However, the main finding was consistent and early association between low sFRP3 levels and higher adiposity, glycemic dysregulation, inflammation and atherogenic lipid ratio in women with GDM. Some examples are presented in **Figure 3B** showing correlations in women with GDM at 14–16 weeks, while no association was seen in normal pregnancies. These associations were in general not present after 30 weeks.

Finally, to evaluate the predictive capacity at 14–16 weeks, we performed ROC curve and logistic regression analyses (**Figure 3C**). For GDM prediction (left panel), clinical indices (TG/HDL, BMI, and ISSI2) showed significant predictive performance, alongside sFRP3 (AUC = 0.33, p<0.01) and DKK1 (AUC = 0.34, p<0.01). ISSI2, sFRP3 and DKK1 are low in GDM and AUC values below 0.5 and Odds Ratios (OR) below 1 reflect an inverse association, with lower biomarker levels indicating greater GDM probability. In multivariable logistic regression (middle panel), both Wnt antagonists remained independently associated with GDM after adjustment for the strongest clinical predictors (ISSI2 and TG/HDL). In the first model, lower DKK1 was associated with higher GDM risk (OR = 0.49 [0.30–0.81], p=0.005) and a similar association was seen for low sFRP3 in the second model (OR = 0.45 [0.23–0.88], p=0.020). For LGA prediction (right panel), maternal BMI, fasting glucose, and ISSI2 were significant predictors, whereas Wnt antagonists and TG/HDL did not predict LGA risk.

### Regulation of Wnt pathways during pregnancy

**Figure 4:**
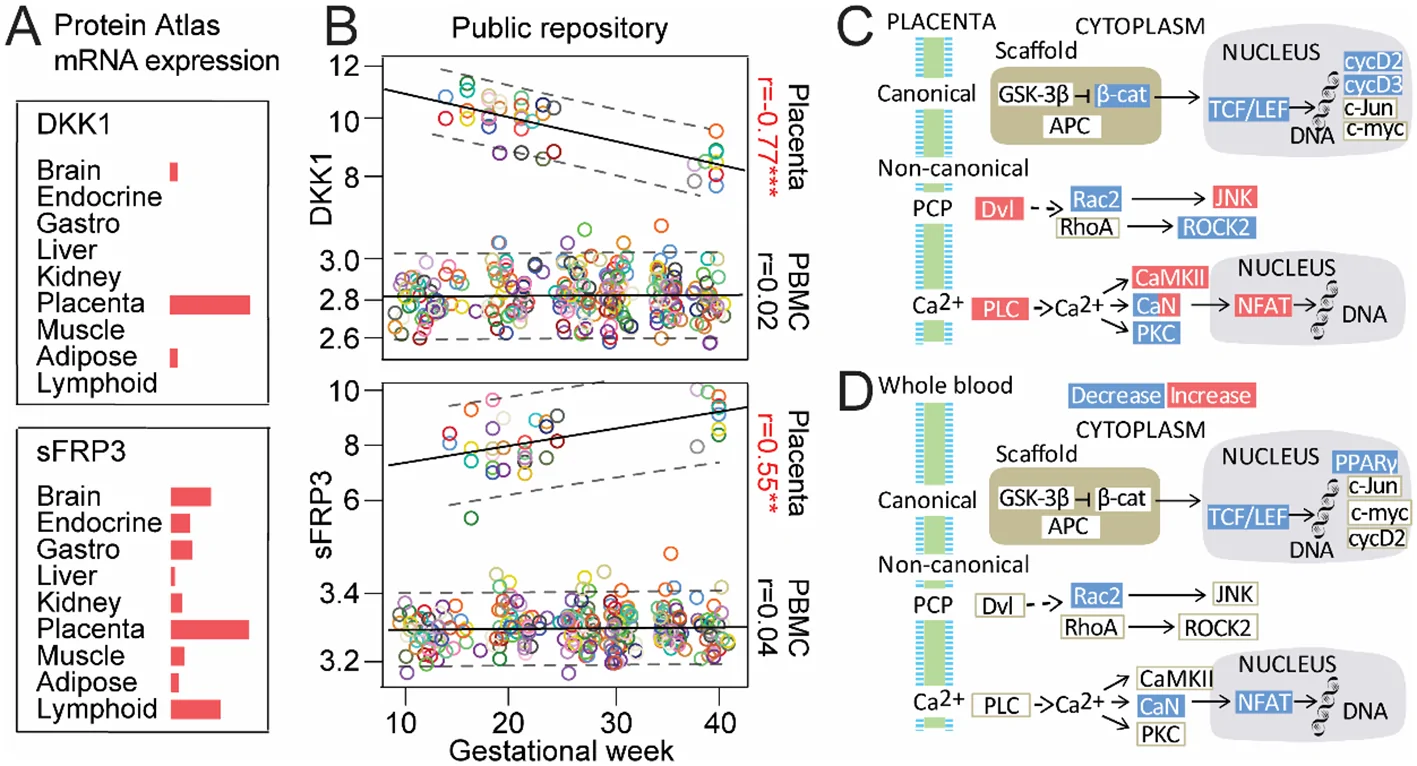
Regulation of Wnt pathways during pregnancy, data from public repositories. **(A)** Tissue expression data on dickkopf-related protein 1 (DKK1) and secreted frizzled related protein 3 (sFRP3) from human protein atlas (proteinatlas.org). **(B)** Correlation between placental (n=36, GSE5999) and maternal whole blood (n=261, GSE121974) mRNA expression of DKK1 and sFRP3 and gestation time in normal pregnancies. For placental samples, correlation with time was assessed by spearman. For PBMC, there were 3–4 samples per woman and partial correlation, adjusting for individual, was performed. **p<0.01, ***p<0.001. Similar to panel B, time-dependent changes in downstream Wnt pathway gene expression (blue, decrease; red, increase) in **(C)** whole blood and **(D)** placenta during normal pregnancy. The image is based on the Wnt signaling pathway in the KEGG database.

To find context for the temporal changes in plasma Wnt modulators, we probed regulation of Wnt pathways during normal pregnancy from available public repositories focusing on placental expression and maternal whole blood (35, 37). First, assessment of mRNA expression from the protein atlas confirmed a high expression in the placenta of *DKK1* and *FRZB* (coding for sFRP3) (**Figure 4A**). Second, during gestation a decrease in *DKK1* and increase in *FRZB* expression was observed in placental biopsies with no change in maternal peripheral blood mononuclear cells (PBMC) (**Figure 4B**). **Figure 4C** shows regulation of the Wnt pathways as outlined by KEGG (Kyoto encyclopedia of genes and genomes) according to gestational age in placental biopsies. The overall impression is a decrease in canonical Wnt signaling as reflected by attenuated *TCF* and transcriptional targets with increasing gestation. Conversely, an increase in important regulators of both non-canonical pathways such as *PLC* and *DVL*, as well as downstream targets such as *JNK* and *NFAT* were observed with increasing gestation. In whole blood, a modestly attenuated Wnt activation was seen during gestation as evaluated by downstream readouts of canonical Wnt signaling such as *TCF7* and *NFAT* reflecting the non-canonical Wnt/Ca2+ pathway (**Figure 3C**).

### Secreted Wnt antagonists at 5-year follow-up

**Figure 5:**
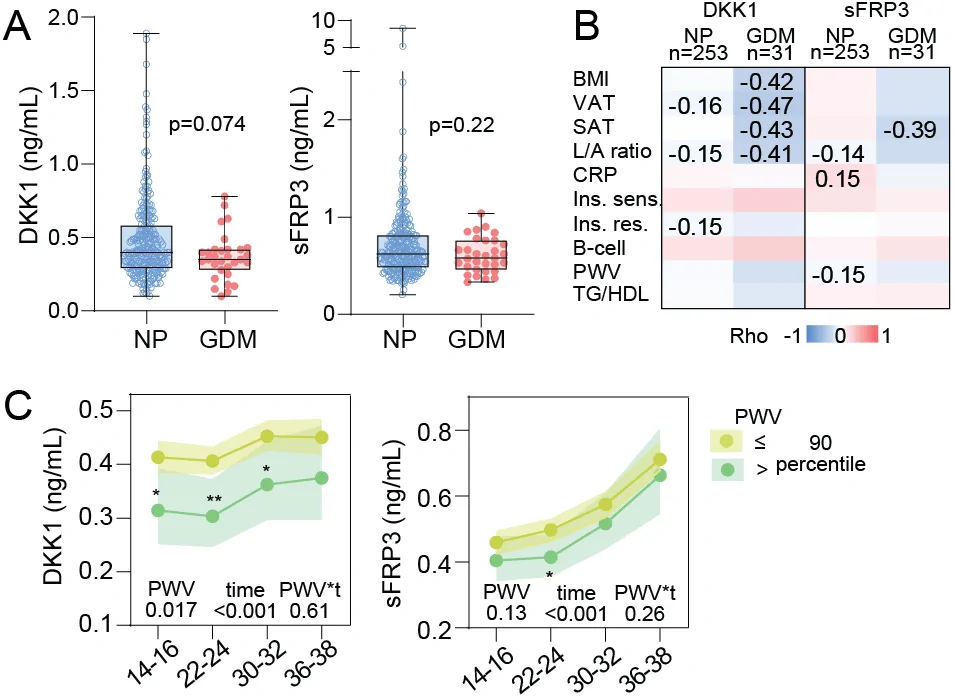
Secreted wnt antagonists at 5-year follow-up. **(A)** Box and whisker plots of plasma levels of Dickkopf-related protein 1 (DKK1) and secreted frizzled related protein 3 (sFRP3) at 5-year follow-up after normal (NP) and pregnancy with gestational diabetes mellitus (GDM). The p-value is from the multivariate GLM with protein as dependent, group (NP or GDM) as fixed and age and BMI as covariates. **(B)** Heatmap showing correlation (spearman, significant correlations are indicated by numbers (rho)) between plasma dickkopf-related protein 1 (DKK1), secreted frizzled related protein 3 (sFRP3) and measures of adiposity (BMI, body mass index; visceral [VAT] and subcutaneous adipose tissue mass [SAT] from whole-body dexa), inflammation (CRP, C-reactive protein; L/A ratio, leptin/adiponectin), glucose metabolism (insulin sensitivity, Matsuda index; insulin resistance, HOMA-IR; β-cell function, insulin secretion-sensitivity index-2 (ISSI2)), pulse-wave velocity (PWV), and atherogenic lipid ratio (TG/HDL, triglycerides/HDL) at 5-year follow-up. **(C)** Temporal profile of DKK1 and sFRP3 according to dichotomized PWV at 5-year follow-up. Data are shown as estimated marginal means and 95% confidence intervals (shaded) from a linear mixed model analysis with age and BMI as covariates. The group effect (PWV), time effect and PWV*time interaction p-values are shown. * p<0.05, **p<0.01 between groups at given time-point.

Finally, we investigated the regulation of DKK1 and sFRP3 and association with metabolic indices at 5-year follow-up. As shown in **Figure 5A**, no significant regulation was observed at follow-up, although there was a trend for lower DKK1 levels in women with previous GDM (p=0.074). The lower DKK1 levels correlated with enhanced adipose tissue mass including VAT as well as with higher leptin/adiponectin ratio reflecting adipose tissue inflammation, in women with previous GDM (**Figure 5B**).

We have previously demonstrated higher aortic stiffness in these women with previous GDM, and while the Wnt antagonists were not related to PWV at follow-up, low levels of DKK1 during pregnancy, independent of GDM status, were associated with having high PWV at follow-up defined as above the 90^th^ percentile (**Figure 5C**) (6). A similar, but less distinct, association was seen for sFRP3.

## Discussion

We investigated circulating modulators of Wnt signaling in women with GDM in relation to different cardio-metabolic indices during pregnancy and long-term follow-up. Our main findings can be summarized: i) plasma DKK1 was lower throughout pregnancy in women with GDM but correlated poorly with metabolic indices. ii) sFRP3 was lower early in pregnancy in women with GDM and correlated with indices of obesity and adipose tissue related inflammation, insulin resistance and poor β-cell function and high TG/HDL ratios. iii) Despite being independently associated with GDM at 14–16 weeks after adjustment for selected clinical indices, the modest AUCs of DKK1 and sFRP3 suggest they give limited clinical information. iv) low levels of DKK1 during pregnancy were associated with indices of higher CVD risk as evaluated by aortic stiffness at 5-year follow-up. Taken together, low plasma sFRP3 during early pregnancy is associated with metabolic and inflammatory dysregulation while persistently low DKK1 may be associated with future CVD risk in GDM pregnancies.

A major and novel finding in our study was the lower sFRP3 levels in women with GDM during early pregnancy. sFRP3 seems to be highly expressed in placental tissue and we recently identified sFRP3 as one of the most abundant proteins released from the placenta into the maternal circulation in healthy pregnancy (22). When assessing overall expression of the Wnt pathways in data obtained from basal plate biopsy specimens from normal pregnancies covering a wide gestational age, canonical Wnt signaling seems more prevalent during early placentation. This is consistent with studies showing an important role of Wnt/β-catenin signaling during implantation and early placental development and with low expression of canonical Wnt antagonists such as DKK1 and sFRP3 (38, 39). We found no studies evaluating sFRP3 in GDM placentas, but Cui et al. demonstrated augmented Wnt/β-catenin signaling in GDM placentas in relation to impaired glucose uptake (18). During pregnancy there is a complex expression pattern and delicate balance of Wnt ligands and frizzled receptors promoting development of the placenta (14). Based on these findings, we hypothesize that abnormal placental development in GDM may be linked to faulty Wnt cell signaling, and that this placental dysfunction may be reflected by altered Wnt-related protein levels in the maternal circulation. Indeed, plasma sFRP3 correlated with multiple cardiometabolic markers including CRP as a reliable marker of inflammation early in pregnancy in women, who later developed GDM. A series of critical proliferation and differentiation processes involving the trophoblast characterize early pregnancy (40). Metabolic and inflammatory insults at the beginning of pregnancy may have long-term effects on placental development. Placental dysfunction is believed to impact placental secretome causing systemic effects on maternal physiology (41). In T2DM, decreased systemic and muscle expression of sFRP3 correlated with increased inflammation and insulin resistance and addition of sFRP3 to myotubes enhanced insulin signaling *in vitro* (27). While there is limited data on sFRP3 in metabolic disease, dysregulated Wnt signaling has been linked to obesity, hyperlipidemia and T2DM (15, 42). Taken together, sFRP3 was associated with a range of cardiometabolic indices and inflammation, restricted to early pregnancy and preceding a GDM diagnosis.

DKK1 is also expressed and abundantly released from placental tissues during normal pregnancy (22), potentially influencing maternal vascular health. In a study of 35 women with GDM and 36 with normal glucose tolerance, no differences in DKK1 or SOST were detected (43). This could be due to the use of IADPSG criteria, rather than our study which utilized the WHO 1999 criteria, as these are associated with more severe metabolic impairment and cardiovascular risk (6). Consequently, translating these findings to cohorts diagnosed under current IADPSG/WHO 2013 criteria require caution; because current criteria capture milder cases of glucose intolerance, the strong early-pregnancy associations observed in our more metabolically impaired cohort may be attenuated in a broader, less severe population. However, transcriptome-wide profiling identified lower DKK1 mRNA expression in GDM placentas (44) and a decrease in DKK1 was associated with placental lipid accumulation in an obesity prone rat model (45). Notably, we have shown increased plasma DKK1 in T2DM (25), which may seem at odds with the current study. However, in contrast to sFRP3, DKK1 levels were not compellingly associated with cardiometabolic indices during pregnancy. The temporal decrease in placental DKK1 expression in biopsies from normal placentas during gestation and increase in plasma levels could reflect an increasing influence from maternal tissues on systemic levels. DKK1 is directly regulated by the β-catenin/TCF complex under physiological circumstances and could partly reflect enhanced canonical Wnt signaling (46), further complicating interpretation of systemic levels.

While we were unable to link the decreased DKK1 levels to any cardiometabolic features during pregnancy, low levels were associated with increased aortic stiffness at 5-year follow-up. In support of our findings, low circulating DKK1 has been associated with increased abdominal aortic calcification in elderly women (47), which has been associated with presence and severity of atherosclerosis and calcification in other vascular beds (48). Arterial function is modified during pregnancy and increased arterial stiffness has been shown from the first trimester, preceding the clinical onset of GDM (49). Indeed, activation of canonical Wnt pathways has been implicated in the development of arterial stiffness (50) and DKK1 expression was attenuated at sites of aortic calcification in Msx2 transgenic mice (51). Pregnancy has been viewed as a stress test where vulnerability to future CVD and metabolic disease may be temporarily unmasked. We speculate that altered activation of canonical Wnt pathways early in pregnancy, as reflected by low plasma DKK1, could relate to vascular alterations that may manifest as CVD later in life, beyond the 5-year follow-up. Moreover, whereas DKK1 levels at follow-up were not associated with aortic stiffness, low levels of DKK-1 at this time point were associated with higher VAT mass and systemic inflammation. Canonical Wnt activation and low DKK1 levels has been linked to hypertrophic obesity (52) and adipose tissue inflammation has been linked to arterial stiffness (53) suggesting interactions between adipose tissue and Wnt signaling could contribute to CVD later in life in these women.

Strengths of this study include a well characterized population-based cohort, a longitudinal design capturing metabolic changes via early (14–16 weeks) and late (30–32 weeks) OGTTs, and similar follow-up time between the GDM and non-GDM groups. Limitations include a relatively low number of women with GDM and the observational nature of the study, limiting us to speculate on mechanisms underlying the findings. We used serum assessments of glucose, rather than fluoride-oxalate plasma, which is preferred for immediate inhibition of glycolysis. This may have introduced some preanalytical reduction in glucose concentrations which could affect GDM classification and calculation of HOMA-IR, the Matsuda index, and ISSI-2. However, the same collection protocol was applied across participants, which reduces concern about differential bias between groups. Additionally, the diagnostic OGTT was performed at 30–32 weeks rather than the standard 24–28 week window used in current guidelines, which may limit direct comparisons and potentially affect case ascertainment. However, this late OGTT effectively captured peak insulin resistance, while the early OGTT minimized the risk of missing high-risk cases. Furthermore, because multiple comparisons were performed without adjustment in this small GDM group, these exploratory associations, both positive and negative, are unstable, sensitive to outliers, and must be interpreted with caution due to limited power. Also, following the absence of GDM-specific differences, we excluded SOST and RSPO3 from downstream correlations to avoid multiple comparison issues and uninterpretable metabolic associations. Still, the potential role for these proteins cannot be ruled out; it simply suggests their involvement is not reflected in circulating plasma levels. We previously observed placental release of secreted Wnt modulators at scheduled cesarean delivery in healthy pregnancies (1). However, we lack data on the contribution of the placental circulation to the systemic levels of the measured Wnt antagonists throughout pregnancy and in GDM, meaning we cannot definitively confirm whether the observed systemic differences stem directly from altered placental regulation. Thus, to get context for the temporal changes in plasma Wnt modulators, we probed data from public repositories to evaluate gestational changes in maternal and placental expression of Wnt signaling members during normal pregnancy. Still, because these repository datasets derive from normal pregnancies, they provide general developmental context but cannot verify GDM-specific placental mechanics. Interpretation of differentially expressed genes from signaling pathways is challenging since intracellular components are shared by multiple pathways and possibly more dependent on phosphorylation status than transcription levels. Finally, although the insulin sensitivity has been validated against the euglycemic-hyperinsulinemic clamp and the β-cell function against the disposition index from the intravenous GTT, these are estimates, and therefore a limitation of the study.

Our findings provide a rationale for future mechanistic studies exploring whether altered placental or systemic Wnt signaling contributes to early metabolic dysfunction in GDM. Specifically, further research could examine if and how genetic variations, microRNA networks, and non-genetic factors influence regulation of DKK1 and sFRP3 in this condition.

In summary, low plasma sFRP3 early in pregnancy is associated with metabolic dysregulation while low plasma DKK1 may be related to future CVD risk as evaluated by aortic stiffness at 5-year follow-up in GDM pregnancies. Although both sFRP3 and DKK1 were independently associated with GDM risk at 14–16 weeks, their low AUC values limit their clinical utility.

## Data Availability

The datasets generated and/or analysed during the current study are not publicly available due to ethical restrictions from the Regional Committee for Medical and Research Ethics in South‐East Norway but are available from the corresponding author on reasonable request. Requests to access the datasets should be directed to tove.lekva@ous-research.no.
